# Effectiveness of COVID-19 vaccines among children 6–11 years against hospitalization during Omicron predominance in Malaysia

**DOI:** 10.1038/s41598-024-55899-5

**Published:** 2024-03-08

**Authors:** Vivek Jason Jayaraj, Masliyana Husin, Jing Lian Suah, Peter Seah Keng Tok, Azahadi Omar, Sanjay Rampal, Sheamini Sivasampu

**Affiliations:** 1 Sector for Biostatistics & Data Repository, National Institutes of Health, Ministry of Health Malaysia, Selangor, Malaysia; 2 Institute for Clinical Research, National Institutes of Health, Ministry of Health Malaysia, Selangor, Malaysia; 3 Data, Analytics and Research, Central Bank of Malaysia, Kuala Lumpur, Malaysia; 4 Department of Social and Preventive Medicine, Faculty of Medicine, Centre for Epidemiology and Evidence-Based Practice, Universiti Malaya, Kuala Lumpur, Malaysia

**Keywords:** COVID-19, Vaccine effectiveness, Hospitalization, Malaysia, Health care, Medical research

## Abstract

There is currently limited data on the effectiveness of COVID-19 vaccines for children aged 6–11 years in Malaysia. This study aims to determine vaccine effectiveness (VE) against COVID-19-related hospitalization after receipt of one- and two-doses of BNT162b2 mRNA (Comirnaty-Pfizer/BioNTech) vaccine over a duration of almost 1 year in the predominantly Omicron period of BA.4/BA.5 and X.B.B sub lineages. This study linked administrative databases between May 2022 and March 2023 to evaluate real-world vaccine effectiveness (VE) for the BNT162b2 mRNA (Comirnaty-Pfizer/BioNTech) vaccine against COVID-19-related hospitalization in the Omicron pre-dominant period with BA.4/BA.5 and X.B.B sub lineages. During the Omicron-predominant period, the cumulative hospitalization rate was almost two times higher for unvaccinated children (9.6 per million population) compared to vaccinated children (6 per million population). The estimated VE against COVID-19 hospitalization for one dose of BNT162b2 was 27% (95% CI − 1%, 47%) and 38% (95% CI 27%, 48%) for two doses. The estimated VE against hospitalization remained stable when stratified by time. VE for the first 90 days was estimated to be 45% (95% CI 33, 55%), followed by 47% (95% CI 34, 56%) between 90 and 180 days, and 36% (95% CI 22, 45%) between 180 and 360 days. Recent infection within 6 months does not appear to modify the impact of vaccination on the risk of hospitalization, subject to the caveat of potential underestimation. In our pediatric population, BNT162b2 provided moderate-non-diminishing protection against COVID-19 hospitalization over almost 1 year of Omicron predominance.

## Introduction

Seroprevalence studies estimate that up to 80% of children in LMICs have had a history of at least one SARS-CoV-2 infection^1^. COVID-19 typically causes mild symptoms and fewer deaths in children compared to adults. Nevertheless, children remain at risk of SARS-CoV-2 infection and may transmit the virus to others, especially within households and schools^2^.

Malaysian epidemiologic data indicate that children constitute 4–8% of all reported cases^3^. This hospitalization rate among children reached its peak during Delta pre-dominance, ranging between 2 and 2.5 per 100,000 population. This figure later decreased by half to 1.5 per 100,000 as the Omicron variant became predominant^4^. These evolving trends suggest a reduction in severity of SARS-CoV-2 infections, during the Omicron wave in contrast to the Delta wave, attributed to advances in therapeutics and case management, increases in populations immunity from previous infection and vaccines, and possibly decreased virulence.

Malaysia’s COVID-19 epidemic and vaccination trends mirrored global dynamics observed during the pandemic with a phased implementation of the national vaccination programme. In the first phase, the Ministry of Health Malaysia’s vaccination strategy targeted maximum coverage for all adults and health workers, which resulted in high uptake of primary and booster doses of vaccines approved for use in adults aged 18 years and above.

In its prioritization strategy, the Ministry of Health’s vaccination programme for children dubbed PICKids carefully took into account epidemiological and clinical evidence, vaccine availability, and operational constraints before expanding the COVID-19 vaccination to include the pediatric population^5,6^. In February 2022, upon authorization of COVID-19 vaccines for use in children, Malaysia provided the BNT162b2 mRNA (Comirnaty-Pfizer/BioNTech) vaccine, administered in two doses of 10 μg, eight weeks apart, for children aged 5–11 years. Initial authorization was based on a demonstration of efficacy using a phase II/III randomized trial for testing BNT162b2 in 5–11-year-olds^7^. Alternatively and subsequent to this authorization, an inactivated whole-virion SARS-CoV-2 vaccine (CoronaVac-Sinovac) was also approved for administration when the BNT162b2 vaccine was contraindicated or at the request of the parents^5^. Post-approval studies have affirmed vaccine safety within children aged 5–11^8^.

Vaccinating children against the Omicron BA.1/BA.2 SARS-CoV-2 infection with any vaccine has been shown to have approximately 50% vaccine effectiveness (VE) against infection and 70% VE against hospitalization^9^. There is a notable decline in protection against infection over a span of two to three months^10–12^. Short-term evaluation of VE against hospitalization during the BA.1/BA.2 peak demonstrated effectiveness ranging from 50 to 80%^8,11,13,14^ with evidence of diminishing VE over a prolonged evaluation period^15^.

As the Omicron variant evolves against a backdrop of widespread immunity in the population, there are still questions about how effective vaccines are in supporting the healthcare system and their duration of protection in children. As such, this study aims to determine vaccine effectiveness (VE) against COVID-19-related hospitalization after receipt of one- and two-doses of BNT162b2 mRNA (Comirnaty-Pfizer/BioNTech) vaccine over a duration of almost 1 year in the predominantly Omicron period of BA.4/BA.5 and X.B.B sub lineages in children between the ages of 6 and 11 in Malaysia. This study also aims to assess the potential decline of vaccine-induced immunity, investigating the mediating role of recent infections of within 6 months on vaccine effectiveness.

## Results

**Figure 1:**
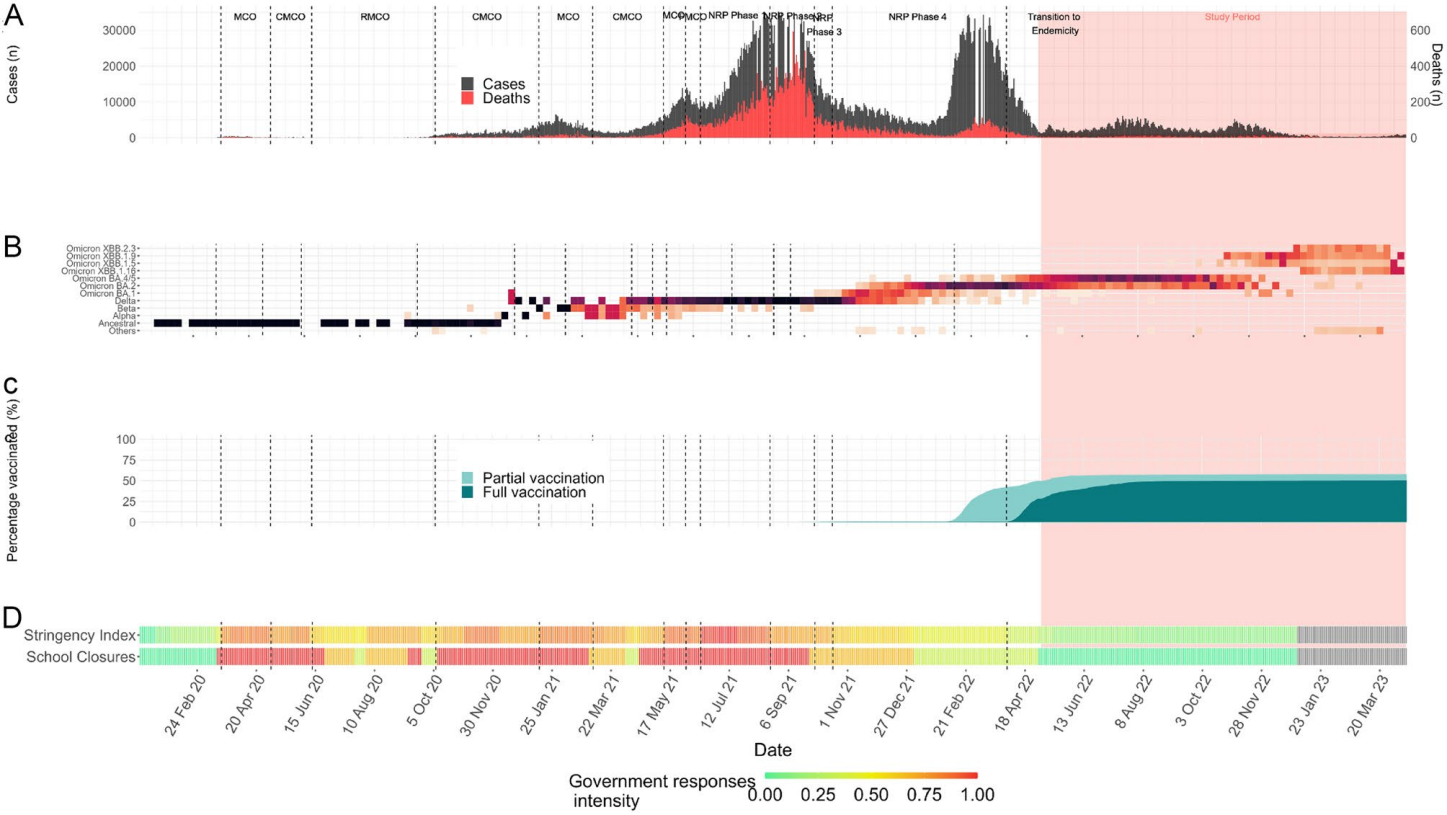
Malaysia’s epidemic curve, variant predominance, vaccination uptake and government responses over calendar date. *MCO* movement control order, *CMCO* conditional movement control order, *RMCO* recovery, movement control order, *FMCO* full movement control order, *NRP* national recovery plan.

Figure 1 presents a multifaceted visualization of COVID-19 trends in Malaysia throughout 2022/2023. Three distinct transmission waves are observed from the epidemic curve, each aligning with the dominance of a new variant: BA.1/BA.2 in early 2022, BA.4/BA.5 by mid-2022, and X.B.B in late-2022. Concurrently, the initiation of vaccinations for children aged 5–11 in February 2022 peaked by early June. Following this, a significant policy shift on 1 April 2022 marked the lifting of all non-pharmaceutical interventions (NPIs), leading to decreased stringency of public health measures and the full reopening of schools.

### Cohort characteristics

A total of 2,474,164 children between the ages of 6 and 11 were studied- of which 1,238,040 were unvaccinated, 136,570 were partially vaccinated with BNT162b2, and 1,099,554 were fully vaccinated with BNT162b2. The unvaccinated group consisted mainly of younger individuals of Malay ethnicity. The study population that received the BNT162b2 vaccine represents the diversity of the pediatric population in Malaysia. The median duration between the first and second doses is 60.7 days (Table 1).

**Table 1:** Baseline characteristics of study cohort and vaccination status.

Characteristic	Overall N = 2474164^1^	Vaccination status			p-value^2^
		Unvaccinated N = 1238040^1^	Partially vaccinated with BNT162b2 N = 136570^1^	Fully vaccinated with BNT162b2 N = 1099554^1^	
Age					< 0.001
6	200,046 (8.1%)	124,803 (10%)	9989 (7.3%)	65,254 (5.9%)	
7	454,873 (18%)	247,958 (20%)	24,763 (18%)	182,152 (17%)	
8	457,304 (18%)	234,285 (19%)	24,939 (18%)	198,080 (18%)	
9	451,910 (18%)	221,276 (18%)	24,890 (18%)	205,744 (19%)	
10	460,834 (19%)	212,734 (17%)	25,628 (19%)	222,472 (20%)	
11	449,197 (18%)	196,984 (16%)	26,361 (19%)	225,852 (21%)	
Male	1,268,584 (51%)	639,505 (52%)	70,700 (52%)	558,379 (51%)	< 0.001
Ethnicity					< 0.001
Malay	1,567,392 (63%)	923,837 (75%)	91,689 (67%)	551,866 (50%)	
Chinese	332,064 (13%)	15,223 (1.2%)	9846 (7.2%)	306,995 (28%)	
Indian	121,706 (4.9%)	71,869 (5.8%)	8202 (6.0%)	41,635 (3.8%)	
Others	453,002 (18%)	227,111 (18%)	26,833 (20%)	199,058 (18%)	
Region^3^					< 0.001
Northern region	484,762 (20%)	262,959 (21%)	26,293 (19%)	195,510 (18%)	
Central region	719,809 (29%)	323,855 (26%)	46,981 (34%)	348,973 (32%)	
Southern region	373,542 (15%)	150,866 (12%)	21,011 (15%)	201,665 (18%)	
Eastern region	418,954 (17%)	297,624 (24%)	16,188 (12%)	105,142 (9.6%)	
Sabah and Sarawak	476,473 (19%)	202,432 (16%)	26,071 (19%)	247,970 (23%)	
Unknown	624	304	26	294	
Duration between vaccines (days)	60.7 (13.8)	–	–	60.7 (13.8)	< 0.001
Infected*	16,491 (0.7%)	4776 (0.4%)	901 (0.7%)	10,814 (1.0%)	< 0.001
History of infection*	101,442 (4.1%)	43,872 (3.5%)	7088 (5.2%)	50,482 (4.6%)	< 0.001
Hospital admission**	688 (< 0.1%)	414 (< 0.1%)	45 (< 0.1%)	229 (< 0.1%)	< 0.001

^1^n (%); Median (IQR).

^2^Pearson’s Chi-squared test; Kruskal–Wallis rank sum test.

^3^Northern region (Perlis, Kedah, Pulau Pinang and Perak), Central region (Selangor, W.P. Kuala Lumpur, and W.P. Putrajaya), Southern region (Negeri Sembilan, Melaka and Johor), Eastern region (Kelantan, Terengganu and Pahang), Sabah and Sarawak (include W.P. Labuan).

*Infected during the study period, History of confirmed infection in last 6 months.

**Hospital admission for COVID-19 during the study period.

### Cumulative risk of COVID-19-related hospitalization within 350 days

**Figure 2:**
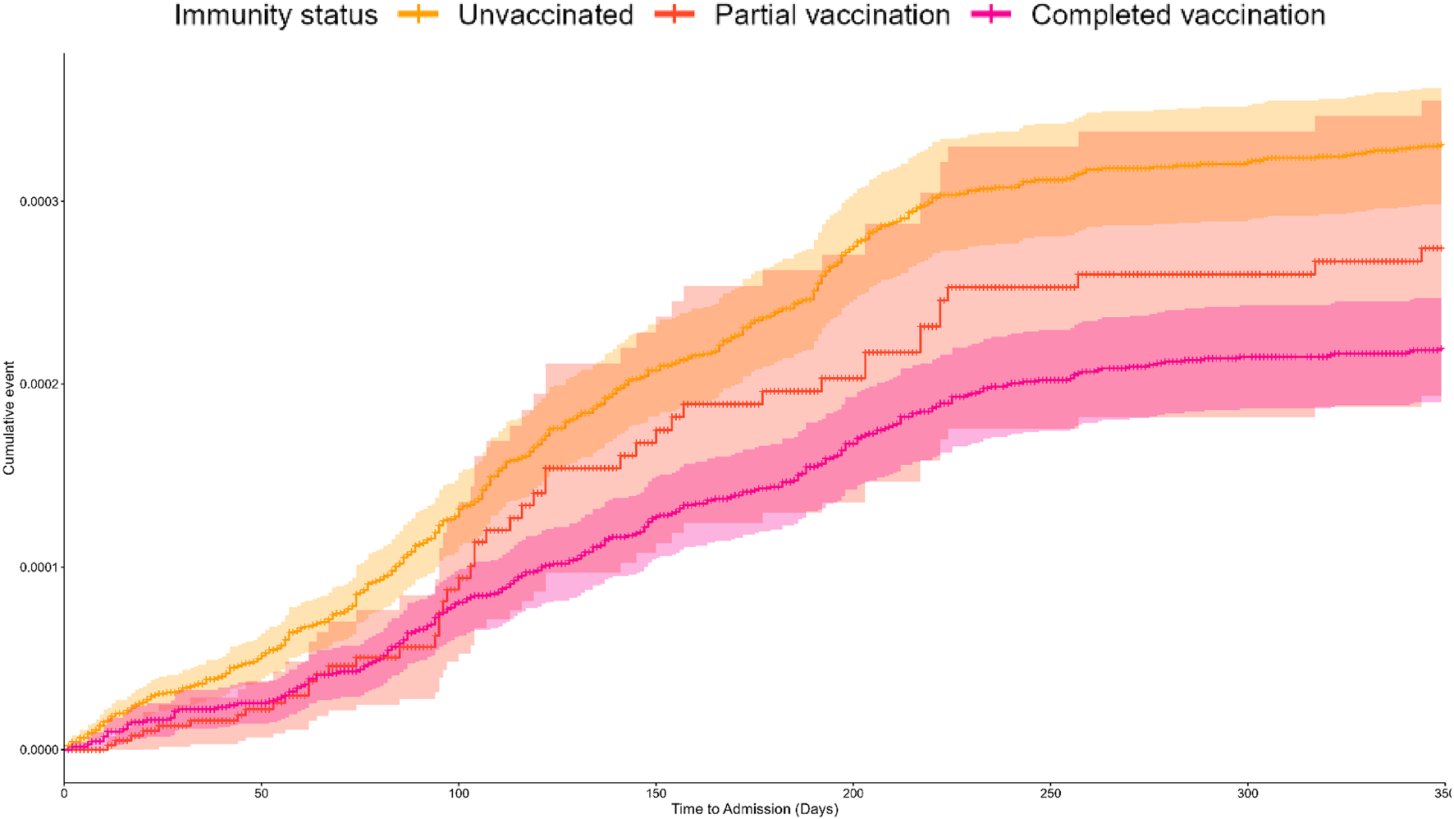
Kaplan–Meier curve showing cumulative incidence (%) and 95% confidence intervals of COVID-19-related hospitalizations by vaccination status.

The Kaplan–Meier curve of COVID-19 hospitalization rates stratified by vaccination status reveals significant differences in cumulative incidences over a 350-day period. An increasing rate of hospitalization is observed, accompanied by a widening disparity between fully vaccinated individuals and those who remain unvaccinated, with apparent divergence at around 50 days post-vaccination. The divergence in hospitalization rates between fully vaccinated and partially vaccinated individuals becomes apparent after 100 days of vaccination, although confidence intervals overlap to some extent (Fig. 2).

### Vaccine effectiveness against COVID-19-related hospitalization over 350-days by dose

A total of 1,238,040 children contributed 431,984,362 person-days, 136,570 children contributed 47,652,852 person-days, and 1,099,554 children contributed 383,697,568 person-days to the unvaccinated, partially vaccinated and fully vaccinated by the end of the follow-up period, respectively (Table 2). Median follow-up time was 242 days (IQR 242–242), in the unvaccinated arm and 127 days (IQR 78–235) in the in the fully vaccinated arm. The variability in the IQR for the fully vaccinated group reflects the dynamic nature of the study design, where individuals transitioned from the unvaccinated to the vaccinated status during the observation period. The overall study cohort had 688 events of COVID-19-related hospitalizations. The cumulative hospitalization rate during the Omicron-predominant period was nearly two times as high among unvaccinated children (9.6 per 10,000,000 population) as among vaccinated children (6 per 10,000,000). The estimated vaccine effectiveness (VE) against hospitalization in children aged 6–11 adjusted for age, sex, region, and history of previous infection was 27% (95% CI − 1%, 47%) and 38% (95% CI 27%, 48%) for one and two doses of vaccines. The adjusted hazard ratio (HR) for those with at least one infection in the last 6 months compared to those with none is 0.88 (95% CI 0.51, 1.23), indicating a 12% reduced risk of COVID-19 hospitalization compared to those without any history of infection. However, this is not statistically significant.

**Table 2:** BNT162b2 vaccine effectiveness against COVID-19 related hospitalization among children 6–11 years old.

	Population	COVID-19 hospitalizations	Follow up time in days (median, IQR)	Person-days	COVID-19 hospitalization incidence rate (per 1,000,000 person-days)	Univariate		Overall		
						HR	95% CI	HR	95% CI	p-value
Vaccination status*										
Unvaccinated	1,238,040	414	242 (242–242)	431,984,362	9.6	Ref	Ref	Ref	Ref	Ref
Partially vaccinated	136,570	45	129 (102–137)	47,652,852	9.4	0.74	0.55, 1.00	0.73	0.53, 1.01	0.06
Completed primary series with BNT162b2 mRNA (Cominarty-Pfizer/BioNTech)	1,099,554	229	127 (78.2–235)	383,697,568	6	0.66	0.54, 0.76	0.62	0.52, 0.73	< 0.01
Recent infection*^a^										
None	2,372,722	668	242 (131–242)	827,935,555	8.1	Ref	Ref	Ref	Ref	Ref
Recent infection within 6 months	101,442	20	132 (76.2–272)	35,399,227	5.6	0.69	0.44, 1.07	0.88	0.51, 1.23	0.61

*HR* hazard ratio, *CI* confidence interval, *Ref* reference category, *IQR* interquartile range.

^a^Recent infectionis defined as any infection within 6 months prior to the study period. A reinfection is defined as an infection after a period of 90 or more days since the previous infection.

*Models were adjusted for age, sex, recent infection within 6 months and stratified by region.

### Vaccine effectiveness against COVID-19-related hospitalization stratified by different period intervals

Vaccine effectiveness (VE) is estimated to be 45% within the first 90 days (95% CI 33, 56%), 47% between 90 and 180 days (95% CI 34, 56%), and 36% between 180 and 350 days (95% CI 22, 45%), all being statistically significant at p < 0.01 (Table 3). Sensitivity analysis of vaccine effectiveness stratified by the number of days after a second dose in children found no clear decline in vaccine effectiveness over time. The interaction model looking at the mediation effect of prior infection on vaccination was found to have a poorer fit than the base model.

**Table 3:** Vaccine effectiveness against COVID-19-related hospitalization among children 6–11 years old at different period intervals.

	Adjusted H.R.	95% CI	p-value
Vaccination status*			
Unvaccinated	Ref	Ref	Ref
Completed primary series (< 90 days)	0.55	0.45, 0.67	< 0.01
Completed primary series (90–180 days)	0.53	0.44, 0.66	< 0.01
Completed primary series (180–350 days)	0.64	0.55, 0.78	< 0.01

*H.R.* hazard ratio, *CI* confidence interval, *Ref* reference category.

*Models were adjusted for age, sex, recent infection within 6 month and stratified by region.

## Discussion

This is the first report of real-world effectiveness of BNT162b2 against COVID-19 hospitalization in children ages 6–11 during a period of almost one year predominated by the BA.4/BA.5 and X.B.B sub-lineages in a low-to-middle income country (LMIC). Completion of a primary series of the BNT162b2 vaccine was estimated to be 38% effective against COVID-19-related hospitalizations in children. This protection persisted over the study period of almost one year, with moderate, non-diminishing effectiveness over time. Our findings also suggest that a recent infection within 6 months does not significantly modify the impact of vaccination on the risk of hospitalization.

Based on our findings, the incidence of COVID-19 hospitalization among children during the Omicron period is observed to be inversely related with the number of vaccine doses received. The incidence rate (IR) among fully vaccinated individuals was 1.5 times lower than that of the unvaccinated group (9.6 vs. 6 per 10,000,000 person-days). Previous studies evaluating the effectiveness of the BNT162b2 vaccine (VE) in the pediatric population against hospitalization predominantly during the BA.1/BA.2 Omicron waves have reported varying results. In the U.S., Price et al.^8^ found a VE of 68% against COVID-19-related hospitalizations, while Shi et al.^14^ estimated the VE to be around 50%. Similarly, in Italy, Sacco et al. reported a 41% VE against severe COVID-19^13^. In Singapore, Tan et al. estimated a very high VE of 85% over a two-month observation period^11^. Nonetheless, a case–control study in Hong Kong estimated the VE of the BNT162b2 vaccine against COVID-19 hospitalizations in children from January to August 2022 to be 44.7% (95% CI 3.4, 68.4%)^15^. While there is a paucity of research focusing on pediatric primary vaccination for the BA.4/BA.5 and X.B.B sub-lineages, our findings and study duration align with those from the latter study in Hong Kong. The comparatively lower VE observed in this study likely stems from heightened community immunity, attributable to multiple infection waves and potential undetected cases, alongside reduced severity in pediatric populations due to physiological differences, such as lower ACE2 expression in nasal pathways^16–18^. Vaccine effectiveness (VE) against hospitalization with the BNT162b2 vaccine without a booster more than 150 days after the last dose in older age groups, was lower than 50%. Similarly, higher VE was observed after administering a booster dose, as indicated by COVID-19 VE pooled data from the I-MOVE-COVID-19 VE network in Europe^19^. Determining superior immunity in children compared to older age groups, or vice versa, poses challenges due to variations in the primary dose timeframe. Additionally, individual risk of exposure to SARS-CoV2 may differ, contributing furhter to this complexity.

Additionally, upon stratification of time intervals, we observed that vaccine effectiveness did not exhibit a distinct decline over time. Our study provides a unique perspective, underscoring the consistent and moderate protection offered by the BNT162b2 vaccine against severe disease, with an effectiveness of around 40% over a year. Our findings are consistent with findings from Hong Kong but with a greater degree of precision, further suggesting moderate non-diminishing effectiveness against COVID-19-related hospitalization in the BA.4/BA.5 and X.B.B Omicron period^15^.

The comparative protection provided by immunity from prior SARS-CoV-2 infection, vaccination and a hybrid of infection and vaccination has been extensively studied in the general population^20,21^. In children, a two-dose BNT162b2 vaccination regimen plus a prior infection (hybrid immunity) has demonstrated protection rates of 74% against Omicron BA.4/BA.5 and 62% against X.B.B. reinfections^22^. In adults, prior infection also provides protection against severe COVID-19 disease. However, no prior studies have explored the impact of hybrid immunity on the risk of COVID-19 hospitalization in children. Our study suggests two findings: a recent infection within 6 months appears to provide some level of protection against hospitalization, aligning with findings from other studies^23^. However, this effect is not statistically significant, possibly due to the small sample with prior infection of approximately 4%, indicating potential under-ascertainment during the Omicron waves or limitations in capturing infections only within the last 6 months. Interestingly, this factor also contributes to our observation that the effects of vaccination on the risk of hospitalization do not appear to be modified by a child’s 6-month history of infection. While there is a possibility that the severity of COVID-19 infection following vaccination remains unchanged in those with a history of previous COVID-19 infection, this notion contradicts other research findings which suggests that hybrid immunity may confer protection against severe COVID-19, particularly in adults from high-income countries^13^. This limitation could have contributed to the lack of statistical significance, necessitating a cautious interpretation of our results. It is worth noting that measuring hospitalization as an outcome in children is different from assessing infection. It is important to consider that various factors can affect hospitalization rates, which may vary by location. Additionally, factors such as hospitalization criteria, bed capacity, and resource limitations must be considered, especially in low- and middle-income countries (LMICs).

Recent studies have indicated that booster doses increased VE to 58.9% in preventing hospitalizations during the BA.4/BA.5 Omicron period^15^. In Malaysia, the Ministry of Health’s PICKids program provides the primary series to all children. It delivers booster doses after 6 to 12 months, prioritizing high-risk groups, including children with significant co-morbidities and those with immunocompromising conditions. In adolescents and older adults, vaccination rates are comparatively high; however, within the pediatric demographic, uptake is less robust, evidenced by approximately 43% receiving primary immunization series and only 0.2% having received booster doses. This disparity could be attributed to a shift in the balance of perceived risks and benefits, including concerns regarding vaccine safety. The approach has shifted from the Delta variant period's more forceful policy of mandatory vaccinations for access to public spaces and events, to the current strategy that relies on appealing to parental responsibility within the PICKids program. This change may reflect growing vaccine hesitancy^24,25^. However, local safety data is reassuring, with 525 reported adverse events following immunization (AEFI) among the targeted age group, equating to 158 reports per million doses, where 94% were non-serious. Of these, 34 reports were of serious AEFIs, including asthma exacerbation and Bell’s palsy, among children aged 5 to 11 years^26^.

Our results pave the way for subsequent investigative efforts. Prolonged surveillance could illuminate the enduring effectiveness of these vaccines, especially in the context of emerging viral variants. As the virus mutates and potentially presents challenges to existing vaccine formulations, there may be a demand for new vaccine compositions specifically targeting these variants. Moreover, the durability of both natural and vaccine-induced immunity may necessitate the exploration of regular booster doses, especially in populations that are more susceptible or exposed to frequent viral challenges. It is a research imperative to continue evaluating the effectiveness, safety, and optimal timing of such boosters. It is essential to delve into the broader implications of these findings on public health policies and vaccination strategies, addressing questions of effectiveness, safety, and optimal timing for booster doses in the Malaysian context.

Our approach used a large dataset with COVID-19 hospitalization as the primary measure. Our study, the largest conducted in low- and middle-income countries (LMICs) to evaluate vaccine effectiveness in children, provides greater precision than previous research. Moreover, as public health worldwide transitions to an “endemic” phase of COVID-19, our study examines the benefits of vaccination amidst a period of economic and social recovery and the relaxation of public health measures. We applied robust methods to address gaps in our knowledge of vaccination programs for younger children, including survival analysis, which allows participants to move between different comparison groups, thereby reducing potential biases related to unmeasured risk behaviours. Our longitudinal data also enabled us to assess how vaccine effectiveness against infection changed over time with a longer follow-up period than any previous study. While our study offers significant insights, it is not without limitations. The observational nature inherently brings potential biases. There could also be inadvertent misclassification of incidental COVID-19 cases during hospitalizations. Such situations might involve cases where patients admitted for unrelated indications with an incidentally positive test misclassified as a COVID-19 hospitalization. This misclassification could result in an underestimation of effectiveness, resembling the estimate for infection rather than accurately reflecting the effectiveness for severe outcome**.** Data linkage could miss matches, which could influence the reliability of estimates. Nevertheless, utilizing high-quality variables like national identification numbers is reinforced by rigorous verification and auditing at the field level, ensuring accuracy and completeness. Accounting for antecedent infections is a critical methodological consideration; however, in the context of the assessment phase and the prevalence of asymptomatic or mildly symptomatic infections, there exists a non-negligible risk of misclassification of prior infection status, a factor of heightened relevance during the Omicron phase. Furthermore, the failure to adjust for pre-existing comorbid conditions may result in the introduction of selection bias into the study results. Ultimately, the dynamics of SARS-CoV-2 epidemiology changes rapidly; hence, the applicability of these findings might be limited to the specific context captured in this study.

## Conclusion

This investigation provides valuable insights into the effectiveness of the BNT162b2 vaccines against COVID-19 hospitalization among children in an LMIC during the BA.4/BA.5 and X.B.B Omicron period. The BNT162b2 vaccine was found to confer moderate protection against children hospitalization with moderate non-diminishing effectiveness over a nearly 1-year period. These estimates are driven by high community immunity within the study period and lower rates of severe outcomes in the study populations. Our findings do not conclusively indicate a protective effect attributable to prior infection or hybrid immunity, suggesting a need for further exploration into the nuances of immune response, with potential constraints arising from under-ascertainment and the focus on infections within the last 6 months Future research should prioritize understanding the long-term protection conferred by vaccines, especially with the advent of newer viral variants and extending the observation period for prior infections. The demand for more efficacious vaccine formulations and the exploration of booster doses will likely emerge as salient areas of study. As global health strategies adapt to the ‘endemic’ phase of COVID-19, understanding vaccine efficacy in younger populations remains pivotal, both for safeguarding individual health and for broader societal protection.

## Methods

### Data sources and outcomes

A cohort of public school-going children aged between 6 and 11 was constructed via the linkage of administrative databases such as student registry, vaccination registry, COVID-19 cases line listing and COVID-19-related hospitalizations. The student registry contains registered and attending public schools under the Ministry of Education (MOE), Malaysia. Vaccination records for the Malaysian population were obtained from the National COVID-19 vaccination register, available via the Vaccine Management System of the Ministry of Health Malaysia (Malaysia Vaccine Administration System, MyVAS). The COVID-19 cases line listing contains information on all COVID-19 confirmed cases and serves as the country’s surveillance system. COVID-19-related hospitalizations were extracted from the Medical Treatment Information System.

We ascertained COVID-19 hospitalisation by primary diagnosis as coded in the hospital as identified by the International Classification of Diseases, Tenth Revision discharge codes U07.1, UO4.9, or B34.2. This includes indication ranging from COVID-19 symptoms (e.g., respiratory symptoms, fever, and dehydration), chest pain, and seizures, whether febrile or not. Additional details can be found in our supplementary material (Supplementary Note 1, Note 2) and have been previously described^27,28^. This constitutes all admissions in public and private hospitals in Malaysia not including emergency care admission. We defined hospitalization using a rule of a length of stay of at least 24 h, and multiple occurrences of hospitalization for one unique individual are considered a new admission if beyond 30 days.

We used deterministic data linkage, relying on the national identifier in all datasets. First, we standardized the identifier by removing non-alphanumeric characters. Exact matches started with the robust student registry dataset. Vaccination data were linked, and non-matches were labelled as non-vaccinated. We linked exposure (vaccination register) and outcomes (Cases and hospitalizations register).

### Definition of variant of concern (V.O.C.) waves

In Malaysia, the distribution of pre-dominant SARS-CoV-2 variants is sequenced and submitted to GISAID EpiCoV database starting from September 2021 and is publicly available. On the basis of this data, we defined three pandemic periods as follows: (1) the Omicron-dominant (BA.1 and BA.2) V.O.C. wave: January to April 2022; (2) the Omicron-dominant (BA.4 and BA.5) V.O.C. wave: May to Sept 2022; and (3) the XBB-dominant V.O.C. wave: Oct to March 2023. This was supplemented with a Bai-Perron sequential breakpoint test on Malaysia’s COVID-19 data, and the estimated Omicron wave was in early February 2022^29^. Figure 1 details the epidemic curve, vaccination uptake, variant predominance and government responses in Malaysia.

### Study design, period and population

**Figure 3:**
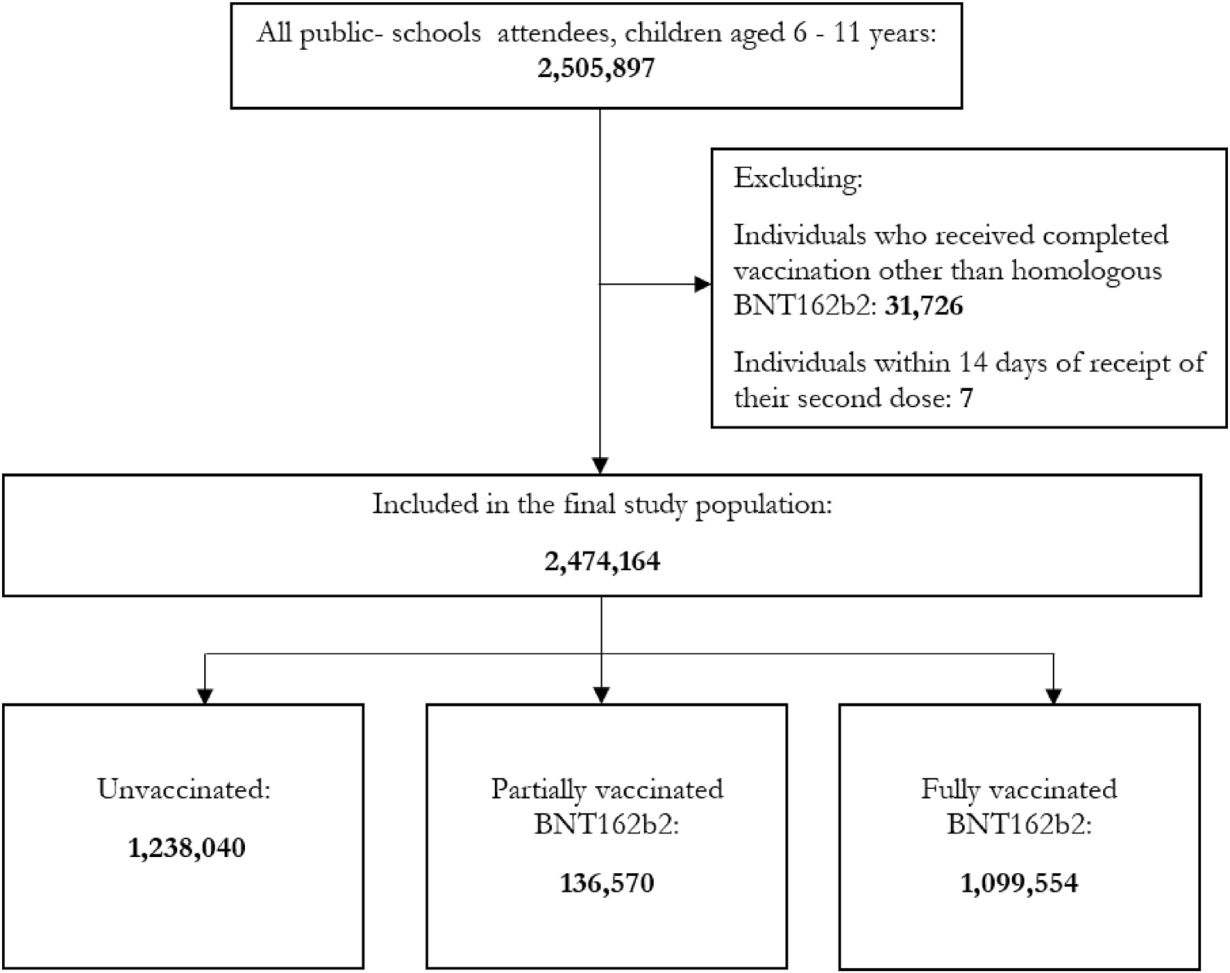
Study participants, 1 May 2022 to 31 March 2023. Participants were between 6 and 11 years of age, public school-going, and vaccinated with at least one dose of BNT162b2. We excluded: (1) individuals who received completed vaccination with other than homologous BNT162b2; (2) individuals within 14 days of receipt of their second dose.

This was a retrospective, population-based cohort study of public school-going children aged between 6 and 11 years in Malaysia. The study population was followed beginning on 1 May 2022 as the Omicron BA.4/BA.5 wave emerged in Malaysia and the vaccination drive in children began peaking. This coincided with a significant policy shift on 1 April 2022 that marked the lifting of all non-pharmaceutical interventions (NPIs), leading to decreased stringency of public health measures*.* The cohort was followed up for a period of 350 days, up till 30 April 2023. We excluded: (1) individuals who received completed vaccination with other than homologous BNT162b2 and (2) individuals within 14 days of receipt of their second dose (Fig. 3). Approximately 97% of children aged 6–11 received homologous BNT162b2, with only a small percentage receiving CoronaVac or other vaccines. Besides ensuring homogeneity among study participants, excluding the small sample of non-BNT162b2 recipients was deemed necessary to prevent potential instability in the VE analysis. Additionally, individuals within 14 days of receiving their second dose were excluded to minimize the inclusion of cases where the protective effects of the vaccine may not have fully developed.

The exposure was vaccination status, such as unvaccinated, partially vaccinated, and fully vaccinated, and it was further stratified by vaccine type. A participant transitions from unvaccinated status to partially vaccinated when he or she receives their first dose. A participant transitions into full vaccination status 13 days after the administration of the second dose. As such, we define each vaccination status as: (1) Unvaccinated days at risk started at the beginning of the study period (1 May 2022) and ended at the time of the occurrence of a study outcome, at the end of the study period, or at the time of receipt of a first dose, (2) Partially vaccinated-days at risk started after receipt of the first dose and ended either at the time of the occurrence of a study outcome, at the time of receipt of second dose or the end of the study period, and (3) Fully vaccinated-days at risk started 14 days after receipt of the second dose and ended either at the time of the occurrence of a study outcome or at the end of the study period. Study participants contributed follow-up time first as unvaccinated persons (before receipt of the first dose) and subsequently as vaccinated persons (after receipt of the second dose). Allowing such crossover in the study design may reduce potential differences arising from unmeasured risk behaviours related to vaccination or infection status.

### Statistical analysis

Transmission, variant, policy and vaccination time trends were assessed visually. The study population was described, and hospitalization risk was estimated for each vaccination stratum. Kaplan–Meier survival curves were employed to visualize and assess risk over time. This provided a non-parametric representation detailing the probability of hospitalization events based on vaccination status and prior exposure to SARS-CoV-2. Following this, Cox proportional hazards regression was applied to compute hazard ratios, contrasting vaccinated with unvaccinated participants while considering recent infections within 6 months. Other selected covariates, due to their apriori importance, included region, age, and sex. The proportionality of hazards was rigorously evaluated using Schoenfeld residuals. Notably, vaccination status and regions deviated from proportionality, prompting the inclusion of vaccination status as a time-varying covariate in the model. Region-induced non-proportionality was handled through stratification. Sensitivity analyses were also conducted, focusing on potential waning of vaccine-induced protection by modelling distinct post-vaccination intervals (3 and 6 months) and probing the mediation effect of recent infection within 6 months on vaccination. Given the differences in testing practices during the alpha and delta periods, where testing was mandatory, we believe that restricting the measurement to a 6-month window provides a more accurate and reliable reflection of the participants’ immune status under a consistent testing policy. All analyses were executed in R, version 4.2.1.

### Ethical considerations

This study was conducted according to the principles of the Declaration of Helsinki. Informed consent was obtained for reporting the diagnosis. This work was conducted as part of The Real-World Evaluation of COVID-19 Vaccines under the Malaysia National COVID-19 Immunisation Programme (RECoVaM) study registered in the National Medical Research Register (NMRR-21-1660-60697). Ethical approval was granted by the Medical Research and Ethics Committee (MREC), Ministry of Health, Malaysia.

### Supplementary Information

Supplementary Information.

## Data Availability

The datasets used and/or analyzed during the current study are available from the corresponding author upon reasonable request.
