# Supplementary material for: Effectiveness of COVID-19 vaccines among children 6–11 years against hospitalization during Omicron predominance in Malaysia
Source: Sci Rep. 2024 Mar 8;14:5690. doi: 10.1038/s41598-024-55899-5 (PMC10920657; doi:10.1038/s41598-024-55899-5)
Supplement: Supplementary file 1 — Supplementary Information. [file 41598_2024_55899_MOESM1_ESM.docx]

**Supplementary appendices**

**Effectiveness of COVID-19 vaccines among children 6-11 years against hospitalization during Omicron predominance in Malaysia.**

Vivek Jason Jayaraj^1^-Masliyana Husin^2^, Jing Lian Suah^3^, Peter Seah Keng Tok^2^, Mohd Azahadi bin Omar^1^, Sanjay Rampal^4^, Sheamini Sivasampu^2^

^1^ Biostatistics and Data Repository Sector, National Institutes of Health, Ministry of Health Malaysia

^2^ Institute for Clinical Research, National Institutes of Health, Ministry of Health Malaysia

^3^ Data, Analytics and Research, Central Bank of Malaysia

^4^ Centre for Epidemiology and Evidence-based Practice, Department of Social and Preventive Medicine, Faculty of Medicine, Universiti Malaya, Malaysia

**Supplementary Note 1: COVID-19 Hospitalization criteria**

COVID-19 admission criteria

(Guideline on home monitoring and management of confirmed Covid-19 case at CAC in primary care - third revision, 21 march 2022, Ministry of Health Malaysia: https://covid-19.moh.gov.my/garis-panduan/garis-panduan-kkm/ANNEX-2m-Guideline-on-Home-Monitoring-n-Mgt-of-Confirmed-COVID-19-Case-at-CAC-in-Primary-Care-3rd-Rev-21032022.pdf):

1. Persistent fever 3 days and more

2. Respiratory distress/abnormal or difficulty in breathing

3. Lethargic/ reduced level of consciousness

4. Poor oral intake with vomiting or diarrhoea

5. Chest pain

6. SPO2 < 95% on room air

7. Dehydration/ not passing urine > 8 hours

8. Persistent or worsening symptoms of cough/ vomiting/ diarrhoea

9. Seizure (febrile or not)

**Supplementary Note 2: Data Sources**

National-level data were extracted from secondary data sources from the surveillance system at the National Crisis Preparedness and Response Centre (CPRC) under the Ministry of Health (MOH) Malaysia. COVID-19 surveillance data are captured from both public and private healthcare facilities based on the provision of the Prevention and Control of Infectious Diseases Act 1988 (Act 342).

The student registry

contains registered and attending public schools under the Ministry of Education (MOE), Malaysia, which covers more than 60% of the total estimated children population in Malaysia (Department of Statistics Malaysia, 2021). Children attending schools in private institution(s) or institution(s) under other government agencies, dropped out of school, undocumented and stateless were not captured in the analysis.

The data collected includes details about the students’ demographics, school informations such as location and type

The National COVID-19 vaccinations register

Vaccination record for the Malaysian population was gathered from the National COVID-19 vaccination register, which is accessed through the Ministry of Health Malaysia's Vaccine Management System (known as Malaysia Vaccine Administration System or MyVAS). This data included information about patients such as their demographics, pre-existing comorbidities, vaccination dates, vaccination sites, vaccine batch numbers, and vaccine types. When patients registered for the COVID-19 vaccine through PICK (an online registration system), they provided background information themselves as a first step. Healthcare providers at vaccination centers reported vaccination-related data through MyVAS. To ensure data accuracy, certain verification steps were implemented. First, authorized personnel at vaccination centers verified the information provided by patients, including vaccine administration details and demographics, using MyVAS. Second, patients were required to scan QR codes at different stations to confirm the vaccine administration through the MySejahtera contact tracing application, which is used for tracking COVID-19 cases in Malaysia. For individuals not using MySejahtera, vaccination center staff performed the verification using MyVAS. The vaccination register covered all individuals who received a COVID-19 vaccine through PICK and served as the authoritative source of information about vaccine recipients in Malaysia. This data was used for planning and implementing the vaccination program, including scheduling appointments, monitoring vaccine coverage across different demographics and risk groups, tracking vaccination rates by location, and resource allocation planning.

The COVID-19 cases line listing

Information regarding COVID-19 confirmed cases was obtained from the national electronic register for COVID-19 cases, which serves as the country's surveillance system for monitoring the disease. This surveillance system gathers data from various sources, including the general surveillance system, entry points into the country, targeted screenings, international health regulation focal points, pre-admission screenings for COVID-19, passive case detection from healthcare facilities, and screenings for deceased individuals (BID). Each patient identified as a confirmed case is given a distinct case number. The data collected includes details about the patients' demographics, location, date of positive test results, the presence of any underlying health conditions, and symptoms reported at the time of diagnosis.

COVID-19 related hospitalizations

COVID-19 related hospitalizations were extracted from the Hospital Discharge Register within the Malaysian Patient Management Information System, Health Informatics Centre, Ministry of Health. The Hospital Discharge Register represents a module within a centralised database known as the Patient Management Information System (Sistem Maklumat Rawatan Pesakit) and has been in operation since 1999. Any hospital admissions where a diagnosis of COVID-19 was present, as identified by the International Classification of Diseases, Tenth Revision discharge codes U07.1, UO4.9, or B34.2, were included, regardless of whether they occurred in a public or private hospital.
